# Evasion of wheat resistance gene *Lr15* recognition by the leaf rust fungus is attributed to the coincidence of natural mutations and deletion in *AvrLr15* gene

**DOI:** 10.1111/mpp.13490

**Published:** 2024-07-02

**Authors:** Zhongchi Cui, Songsong Shen, Linshuo Meng, Xizhe Sun, Yuqing Jin, Yuanxia Liu, Daqun Liu, Lisong Ma, Haiyan Wang

**Affiliations:** ^1^ College of Plant Protection Hebei Agricultural University/Technological Innovation Center for Biological Control of Crop Diseases and Insect Pests of Hebei Province Baoding Hebei China; ^2^ The State Key Laboratory of North China Crop Improvement and Regulation College of Horticulture Baoding Hebei China

**Keywords:** avirulence gene, AvrLr15, *Puccinia triticina*

## Abstract

Employing race‐specific resistance genes remains an effective strategy to protect wheat from leaf rust caused by *Puccinia triticina* (Pt) worldwide, while the newly emerged Pt races, owing to rapid genetic evolution, frequently overcome the immune response delivered by race‐specific resistance genes. The molecular mechanisms underlying the newly evolved virulence Pt pathogen remain unknown. Here, we identified an avirulence protein AvrLr15 from Pt that induced *Lr15*‐dependent immune responses. Heterologously produced AvrLr15 triggered pronounced cell death in *Lr15*‐isogenic wheat leaves. AvrLr15 contains a functional signal peptide, localized to the plant nucleus and cytosol and can suppress BAX‐induced cell death. Evasion of *Lr15*‐mediated resistance in wheat was associated with a deletion and point mutations of amino acids in AvrLr15 rather than *AvrLr15* gene loss in the *Lr15*‐breaking Pt races, implying that AvrLr15 is required for the virulence function of Pt. Our findings identified the first molecular determinant of wheat race‐specific immunity and facilitated the identification of the first *AVR/R* gene pair in the Pt–wheat pathosystem, which will provide a molecular marker to monitor natural Pt populations and guide the deployment of *Lr15*‐resistant wheat cultivars in the field.

## Graphical abstract

Avirulence protein AvrLr15 from *Puccinia triticina* induces *Lr15*‐dependent immune responses; evasion of *Lr15*‐mediated resistance in wheat is associated with a deletion and point mutations of amino acids in AvrLr15.

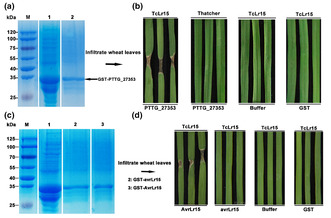

Wheat is one of the most important grain crops, which provides food resources to billions of people. With the world population continuously growing, safeguarding wheat production by reducing disease threats is becoming extremely important. Notably, one of the significant wheat yield losses is associated with the biotrophic fungal pathogen *Puccinia triticina* (Pt), which causes leaf rust disease and leads to epidemics under favourable environmental conditions (Bolton et al., 2010; Huerta‐Espino et al., 2011; Ordoñez et al., 2010).The deployment of resistance (*R*) genes in wheat cultivars that confer race‐specific resistance against Pt isolates is the most effective and environmentally safe approach to reducing the damage caused by leaf rust (Ellis et al., 2014). However, the natural population of Pt isolates is subjected to the continuous and strong selection pressures exerted by race‐specific resistance genes, resulting in the rapid emergence of new Pt isolates with the ability to avoid R gene recognition (Kolmer et al., 2007). Because the interaction between wheat and Pt follows the gene‐for‐gene model, the emergence of resistance‐breaking Pt isolates highlights the urgent need to identify Pt avirulence genes and to decipher the molecular mechanisms underlying their recognition and evasion by *R* genes, which will guide and prioritize the deployment of resistance genes in different geographic regions worldwide.

A pathogen avirulence protein is perceived by its cognate immune receptor, which activates the host immune response that frequently results in localized programmed cell death (PCD) at the site of pathogen infection (Collins et al., 2011; Elmore et al., 2011). To identify an avirulence protein of Pt, we selected a highly up‐regulated effector candidate gene *PTTG_27353* (GenBank accession number: OAV93472.1) from our previous RNA‐seq data (CNGBdb: PRJNA694214) derived from the susceptible wheat cv. Chinese Spring interacting with Pt race PHNT (Cui et al., 2023). To validate the gene expression detected in the RNA‐seq data, the expression profiles of *PTTG_27353* in Chinese Spring inoculated with Pt PHNT were examined by reverse transcription‐quantitative PCR (RT‐qPCR) (Text S1). In comparison with the expression level at 0 hour post‐inoculation (hpi), the expression of *PTTG_27353* was significantly induced and peaked at 12 hpi and subsequently decreased from 24 to 96 hpi (Figure 1a). Previous studies indicated that haustorial formation occurrs at 24 hpi (Prasad et al., 2020; Qi et al., 2018; Yang et al., 2020) suggesting that *PTTG_27353* may be involved in the initial infection of wheat by Pt. Next, we produced the recombinant protein PTTG_27353 (Figure 1b, Table S1) using the *Escherichia coli* expression system (Figure 1c, Figure S1, Text S1) and infiltrated the purified protein into 42 near‐isogenic lines of Thatcher carrying a single leaf rust *R* genes. Cell death occurred in the leaves of line TcLr15 (RL6052) when PTTG_27353 protein was infiltrated, but no cell death was evident in the leaves of the other 41 near‐isogenic lines or in the negative control infiltrated with buffer (Figure S2). To confirm this initial observation, we repeated this experiment three times, and consistent results were obtained (Figure 1d). Therefore, PTTG_27353 induced an *Lr15*‐dependent cell death response in the wheat TcLr15 line, implying that PTTG_27353 is an AvrLr15 candidate (GenBank accession number: OQ458736.1) that can trigger an *Lr15*‐dependent immune response in wheat leaves. Protein‐mediated phenotyping assay in wheat is emerging as a rapid and effective method that facilitates the functional studies of effectors from wheat fungal pathogens (Sung et al., 2021). For example, the infiltration of heterologously produced wheat stem rust effector protein AvrSr35 into *Sr35* wheat leaves triggers strong cell death (Salcedo et al., 2017). In addition, infiltration of effector protein SnTox3 from *Parastagonospora nodorum* induces a strong necrotic response in susceptible wheat, while infiltration of TaPR1 proteins in wheat leaves can suppress *P. nodorum* disease (Sung et al., 2021). To confirm the function of *AvrLr15*, we silenced the expression of *AvrLr15* by BSMV‐host‐induced gene silencing (HIGS) in TcLr15 wheat (Text S1). Compared with the control plants, more urediniospores appeared on the leaves of *AvrLr15*‐silenced plants after inoculation with the avirulent race PHNT (Figure 2). The silencing efficiency, monitored by RT‐qPCR, indicated that the relative expression of *AvrLr15* was significantly reduced by 76% and 72% after infection with avirulent Pt at 24 and 48 hpi, respectively (Figure S3). All these results indicate that *AvrLr15* triggers the disease resistance response of TcLr15.

**FIGURE 1:**
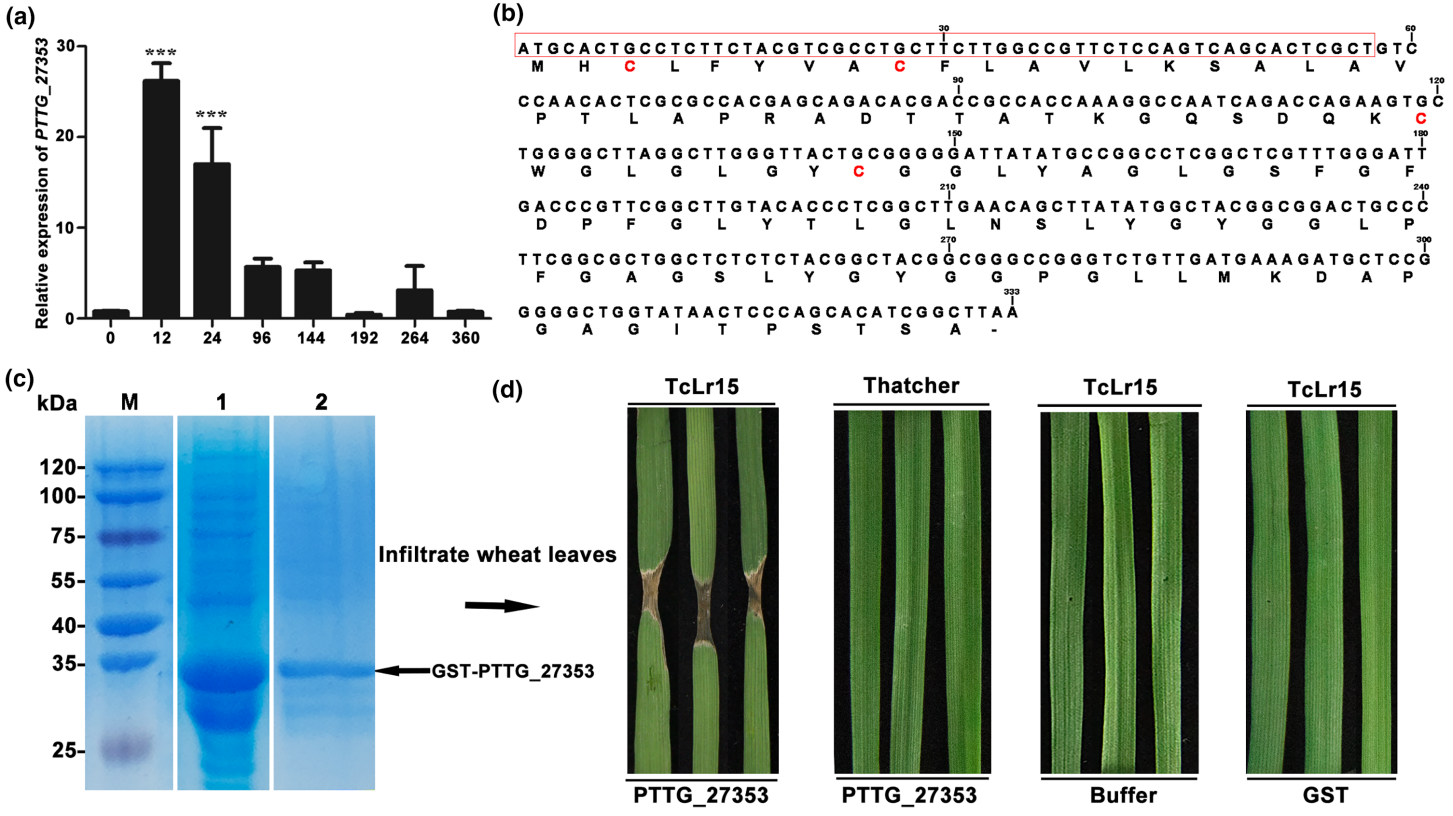
PTTG_27353 effector protein induces *Lr15*‐dependent cell death in the leaves of wheat. (a) The expression profiles of *PTTG_27353* during *Puccinia triticina* infection of the susceptible wheat cultivar Thatcher at different time points. The relative expression of *PTTG_27535* was quantified using reverse transcription‐quantitative PCR. The *y*‐axis indicates the amounts of *PTTG_27535* gene transcript normalized to the *β‐actin* gene. The *x*‐axis indicates sampling times (hours post‐inoculation). Values are means ± *SEM* of three independent experiments. The statistical significance of differences was calculated using a one‐way analysis of variance (ANOVA) and Duncan's multiple range test (DMRT) with *p* < 0.05. For each treatment, three independent biological replicates were used for analysis. (b) Amino acid and nucleotide sequences of PTTG_27353. The red box indicates the signal peptide sequence of PTTG_27353; the cysteine residues are marked in red. (c) SDS‐PAGE analysis showed the IPTG‐induced and purified PTTG_27535 protein. Lane 1: crude protein of PTTG_27353 induced by IPTG. Lane 2: purified protein PTTG_27353. M: protein marker. (d) Infiltration of PTTG_27535 protein into the leaves of TcLr15 and Thatcher wheat lines. Buffer and purified glutathione S‐transferase (GST) protein were used as a negative control.

**FIGURE 2:**
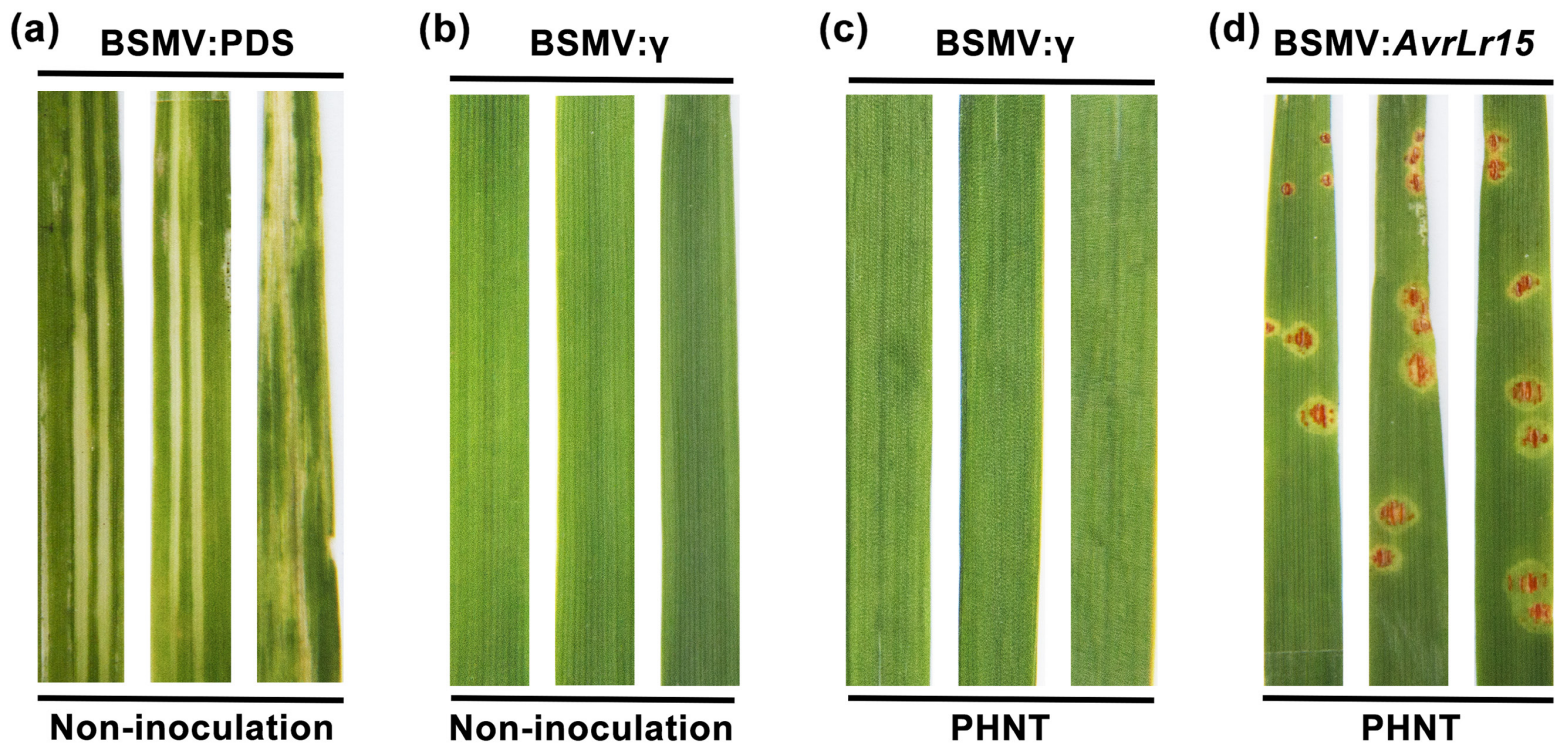
Silencing of *AvrLr15* increases virulence of *Puccinia triticina*. (a) Photobleaching phenotypes were evident on the fourth leaves of plants inoculated by BSMV:PDS at 10 days post‐inoculation (dpi). (b) The leaves inoculated with empty vector BSMV:γ used as the control. (c) The phenotype of wheat leaves inoculated with avirulent pathotype PHNT and infected with BSMV:γ. (d) Disease phenotypes of the fourth leaves were pre‐inoculated with BSMV:*AvrLr15* and infected with the avirulent pathotype PHNT at 10 dpi.

The full‐length *AvrLr15* gene was 420 bp, including two exons and one intron (Figure S4). A primer pair in the untranslated region (UTR) was used to determine the introns and exons of the genomic DNA sequences of different races (Figures S5 and S6, Table S1). Rust fungi from different plant species were examined using PCR, and the results showed that *AvrLr15* could only be detected in wheat leaf rust fungi (Figure S7). The coding sequence of *AvrLr15* encodes a 13 kDa protein corresponding to 110 amino acids, without a known conserved domain and containing four cysteine residues. SignalP v. 5.0 predicted the presence of an N‐terminal signal peptide containing 19 amino acids in the AvrLr15 protein, and effector P‐Fungi v. 3.0 preded that AvrLr15 may be an effector that could be secreted into the host (Figure S8). To examine the functional role of the putative signal peptide (SP) of AvrLr15, the yeast signal sequence trap system (Jacobs et al., 1997) was employed (Text S1). *SP*_*AvrLr15*_ was cloned and ligated to the pSUC2 vector (Figure S9, Table S1). Yeast strains YTK12 transformed with SP_AvrLr15_ or with a construct with the signal peptide of Ps87 (as positive control; Gu et al., 2011) were able to grow on CMD − W and YPRAA media (Figure 3a). 2,3,5‐triphenyltetrazolium chloride (TTC)‐treated Ps87 and SP_AvrLr15_ culture filtrates turned red, whereas the negative control culture filtrates treated with TTC remained colourless (Figure 3a). These results indicated that AvrLr15 does contain a functional signal peptide responsible for the secretion of AvrLr15 into the plant cell. Inhibition of BAX‐induced cell death is often regarded as a criterion for suppressing plant immunity (Tao et al., 2021) (Text S1). _ΔSP_AvrLr15 was cloned and ligated to the pCamA vector (Figure S10, Table S1). To examine the ability of AvrLr15 to inhibit BAX‐induced cell death, we infiltrated *Agrobacterium tumefaciens* carrying pCamA:_ΔSP_AvrLr15 into *Nicotiana benthamiana* and 24 hours later *A. tumefaciens* carrying pEarlyGate100:BAX was infiltrated. Figure 1f shows that _ΔSP_AvrLr15 completely inhibited BAX‐induced cell death in *N. benthamiana* (Figure 3b). The expression of GFP‐tagged _Δ*SP*_ *AvrLr15* in pCamA:_ΔSP_AvrLr15 after agroinfiltration in *N. benthamiana* leaves was confirmed by green fluorescent signals under microscopy; _ΔSP_AvrLr15 localized in the plant nucleus and cytosol (Figure S11). These findings indicate that AvrLr15 was able to inhibit BAX‐induced cell death, suggesting that AvrLr15 has the virulence function in planta.

**FIGURE 3:**
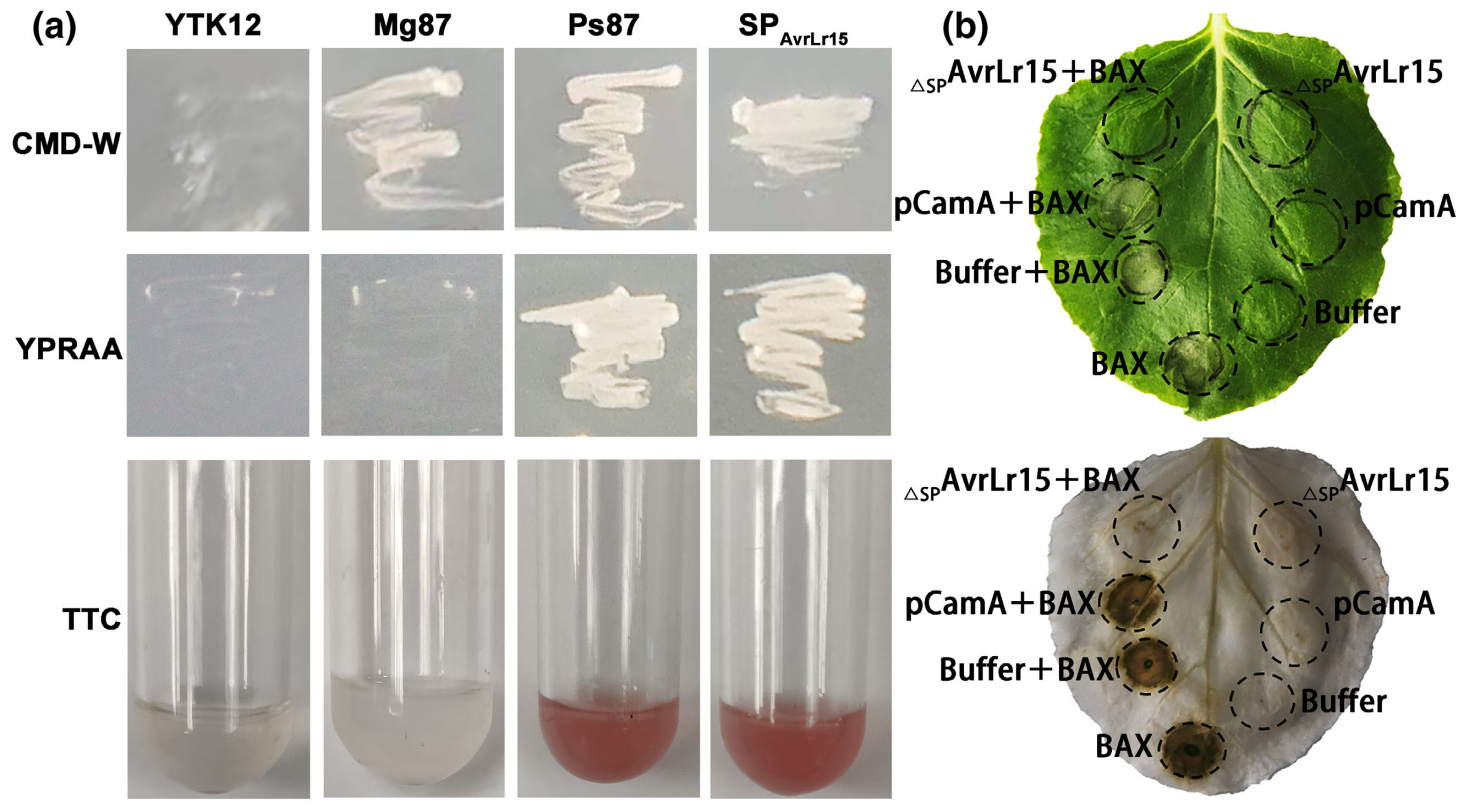
AvrLr15 contains a functional signal peptide and can suppress BAX‐induced cell death in *Nicotiana benthamiana*. (a) Functional validation of the putative N‐terminal signal peptide of AvrLr15 using the yeast invertase secretion assay. The predicted signal peptide coding sequence of AvrLr15 (SP_AvrLr15_) was cloned into the yeast secretion trap vector pSUC2. The signal peptide of Ps87 was used as a positive control, and empty vector and the signal peptide of Mg87 were used as a negative control. Invertase activity was detected with 2,3,5‐triphenyltetrazolium chloride (TTC). (b) Inhibition of BAX‐induced cell death on *N. benthamiana* leaves. *Agrobacterium tumefaciens* carrying _Δ*SP*_ *AvrLr15* was infiltrated into *N. benthamiana* leaves, followed 24 h later by infiltration with *A. tumefaciens* carrying the *BAX* gene. pCamA and buffer served as a negative control. The same leaf was examined before (top) and after (bottom) being decolourized with the destaining solution.

To date, over 80 *Lr* resistance genes conferring race‐specific seedling or adult plant resistance have been named and deployed in wheat against leaf rust (Kuldeep et al., 2022). *Lr15*, as a seedling resistance gene, confers resistance against leaf rust at the seedling stage. With the increased deployment of *Lr15* in the field, the emergence of new *Lr15*‐breaking races has been reported in many wheat‐growing regions worldwide (Dholakia et al., 2013). To understand the mutation mechanism of *AvrLr15*, we amplified the *AvrLr15* genes in 25 natural Pt isolates. PCR amplification products were obtained from these tested isolates and were sequenced. Ten *AvrLr15* gene sequences amplified from *Lr15*‐breaking isolates all contained nucleotide deletions that led to one amino acid deletion located at amino acid residue P92 and multiple nucleotide changes that resulted in three amino acid substitutions at residues P80, M96 and P106 compared to AvrLr15 protein sequences (Figure S12), implying that the virulence allele of *AvrLr15* is highly conserved. To determine whether this virulent *avrLr15* variant could avoid the recognition by *Lr15*, we cloned the virulent *avrLr15* for protein production, and the purified protein was used for infiltration into TcLr15 leaves (Figure 4a). Figure 4b shows that avrLr15 was compromised in inducing cell death in the infiltrated region, similar to the negative controls (buffer and glutathione S‐transferase). Next, we examined the localization of avrLr15 and its ability to inhibit BAX‐induced cell death. _ΔSP_avrLr15 was cloned and ligated to the pCamA vector (Figure S13). GFP‐tagged avrLr15 localized to the plant nucleus and cytosol, similar to the localization of AvrLr15, after agroinfiltration in *N. benthamiana* leaves (Figure S14). Figure S15 shows that _ΔSP_avrLr15 completely suppressed BAX‐induced cell death in *N. benthamiana*, suggesting the virulence allele of *AvLr15* is also required for Pt virulence.

**FIGURE 4:**
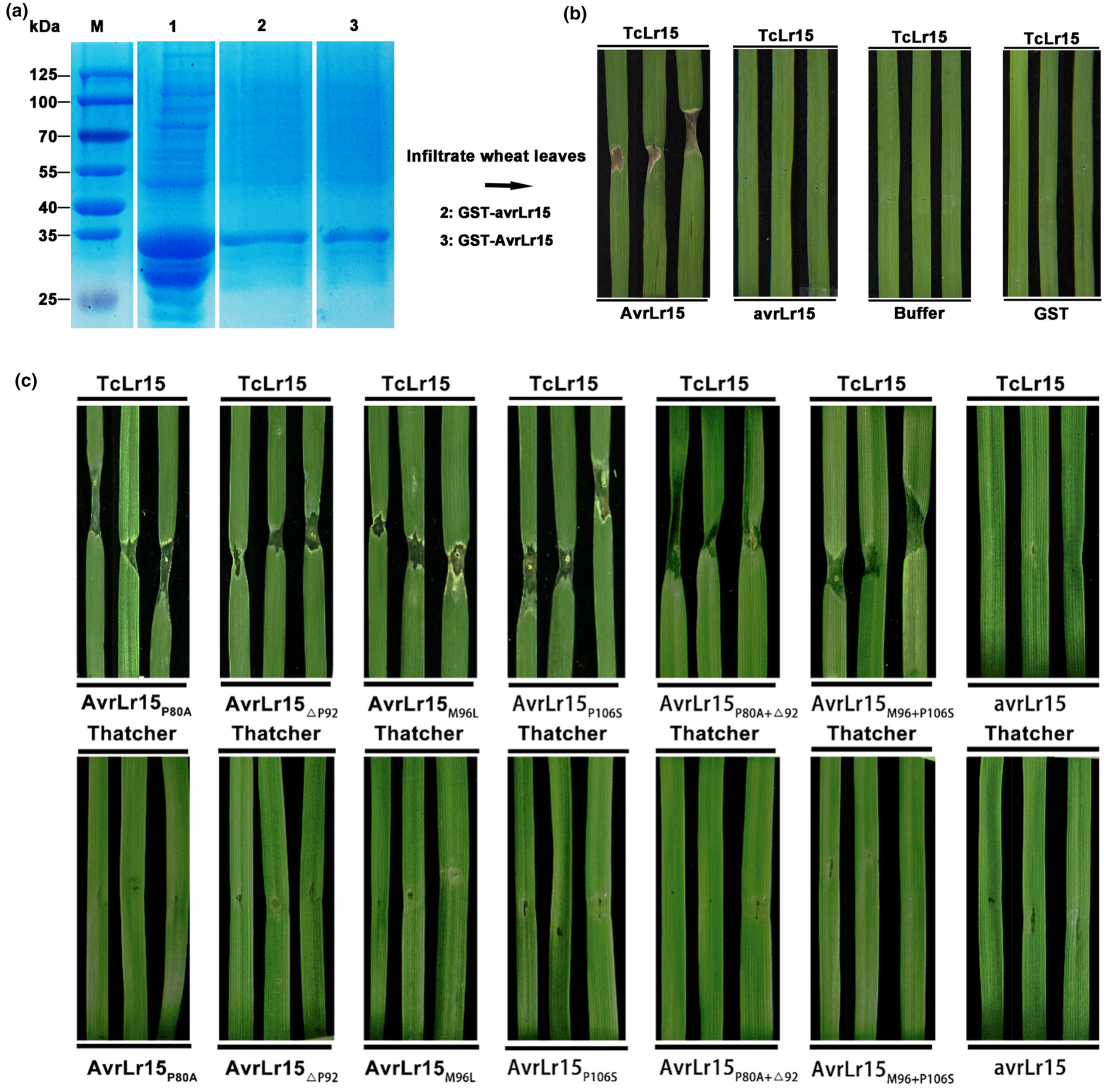
Virulent allele *avrLr15* is not able to induce *Lr15*‐dependent cell death. (a) SDS‐PAGE analysis shows the IPTG‐induced and purified avrLr15 protein. Lane 1: Crude protein of avrLr15 induced by IPTG. Lane 2: Purified protein avrLr15. Lane 3: Purified protein AvrLr15. (b) avrLr15 protein with three amino acid mutations and one deletion are not able to induce *Lr15*‐dependent cell death in TcLr15 leaves. Infiltration of avrLr15 protein into leaves of wheat near‐isogenic line TcLr15. Buffer and glutathione S‐transferase (GST) protein were used as negative controls. Purified protein AvrLr15 was used as a positive control. (c) Four mutants with single amino acid changes (AvrLr15_P80A_, AvrLr15_ΔP92_, AvrLr15_M96L_, AvrLr15_P106S_), and two mutants with double amino acid changes of AvrLr15 (AvrLr15_P80A+ΔP92_, AvrLr15_M96L+P106S_) were able to induce *Lr15*‐dependent cell death in TcLr15 leaves. Infiltration of avrLr15 protein into leaves of wheat near‐isogenic line TcLr15 was used as control.

To clarify which amino acid change affects the recognition of AvrLr15 by Lr15, we constructed four mutants with single amino acid changes (AvrLr15_P80A_, AvrLr15_ΔP92_, AvrLr15_M96L_, AvrLr15_P106S_), and two mutants with double amino acid changes of AvrLr15 (AvrLr15_P80A+ΔP92_, AvrLr15_M96L+P106S_) (Figure S16). The purified mutated proteins were used for infiltration into TcLr15 leaves; avrLr15 was used as a control. The results showed that all the mutated proteins could cause a hypersensitive response (HR) in the leaves of TcLr15, but not in Thatcher wheat lines (Figure 4c), indicating that the loss of avirulence in *AvrLr15* necessitates the concurrent occurrence of four amino acid mutations. The high conservation of avrLr15 variants identified from the *Lr15*‐breaking Pt races suggested natural and direct selection from the *Lr15* gene acted on *AvrLr15* genes in a specific arms race. Gain of virulence through insertion and deletions has recently been observed in wheat stem rust effector proteins. A microtransposon (mite) insertion in the sixth exon of wheat stem rust fungus *Puccinia graminis* f. sp. *tritici* (Pgt) *AvrSr35* resulted in early termination of the protein translation, and which thus overcomes the recognition by the Sr35 immune receptor protein (Salcedo et al., 2017). Functional analyses showed that one residue substitution, Q121K, in the coding sequence of Pgt *AvrSr50* gene was sufficient to escape recognition by Sr50 (Chen et al., 2017). In addition, the deletion of the *AvrSr27* locus in the field *Sr27*‐breaking Pgt clonal lineages has been reported (Upadhyaya et al., 2021). In contrast to amino acid differences in the mature protein, the variant avrSr27‐3, without any sequence changes compared to AvrSr27‐2, was expressed approximately four‐fold lower than the avrSr27‐2 variant, and also led to the lack of an avirulence phenotype. Huang et al. (2019) found that the virulent race of *Phytophthora sojae* does contain the avirulence gene *Avr3C‐1*, but transcription of *Avr3C‐1* could not be detected by RT‐PCR in the virulent race. Similarly, we found one *Lr15*‐breaking race (64‐L‐3) does contain the *AvrLr15* sequence in the genomes of leaf rust isolates from Australia (Wu et al., 2017) (Figure S17). We hypothesized that the resistance‐breaking might be caused by the lack of expression of *AvrLr15*, so it is necessary to look for the expression of *AvrLr15* in virulent races in the future. In addition, extensive nuclear exchange events might occur in natural populations of Pt to generate new gene combinations and pathotypes (Sperschneider et al., 2023). In the current study, we identified Pt avirulence gene *AvrLr15* and clarified that mutations in four amino acids in the AvrLr15 protein lead to the loss of avirulence, which will help develop a molecular marker to monitor Pt races in the field for early detection of *Lr15*‐breaking races. *AvrLr15* could also be used to confirm the expression of the functional *Lr15* protein in the resistance gene cassettes, allowing for *Lr15* to be quickly pyramided alongside other *R* genes. As more *R‐Avr* gene pairs are identified, they can be used to guide the selection of complementary *R* genes targeting multiple avirulence factors to increase the durability of the deployed resistance gene pyramids and reduce the probability of spontaneous virulent Pt strainsa arising.

## CONFLICT OF INTEREST STATEMENT

The authors declare no competing interests.

## Supporting information


**Figure S1.** Detection of recombinant plasmid pGEX‐6P‐3:PTTG_27353 by PCR. (1) The amplification of empty pGEX‐6P‐3 vector; (2) the amplification of pGEX‐6P‐3:PTTG_27353; M, marker.

**Figure S2.** Transient expression of *PTTG_27353* induced a hypersensitive response in wheat TcLr15, a near‐isogenic line of Thatcher. Vector was used as a negative control for infiltration (left leaf of each pair); leaves on the right were infiltrated with PTTG_27353 protein. The red box pinpoints TcLr15.

**Figure S3.** Silencing efficiency assay of *AvrLr15* in AvrLr15‐silenced plants at 24, 48 and 120 hours post‐inoculation (hpi) with *Puccinia triticina*. Wheat leaves inoculated with empty vector BSMV:γ were used as controls.

**Figure S4.** Analysis nucleotide sequence of *AvrLr15*. (a) The red box indicates exons of *AvrLr15*. The green box indicates intron of AvrLr15. Black lines indicate untranslated region (UTR). Arrows indicate primer direction. (b) The red line indicates the exon sequence of the *AvrLr15*. The green line indicates the intron sequence of *AvrLr15*.

**Figure S5.** PCR amplification products from genomic DNA of different *Puccinia triticina* races are shown after separation on a 1% agarose gel. 1, PHTT. 2, PHNT. 3, KHKT. 4, THKT. 5, RHHT. 6, THTT. 7, THTS. 8, sterile water. M, marker.

**Figure S6.** Multiple sequence alignment of *AvrLr15* from different *Puccinia triticina* races. The red line indicates the exon sequence of the *AvrLr15*. The green line indicates the intron sequence of *AvrLr15*.

**Figure S7.** PCR amplification products from different plant species rust fungi races are shown after separation on a 1% agarose gel. 1, *Puccinia triticina*. 2, *Puccinia striiformis* f. sp. *tritici*. 3, *Puccinia graminis* var. *tritici*. 4, *Phakopsora ziziphivulgaris.* 5, *Gymnosporangium yamadai*. 6, *Uromyces verrucosus*. 7, *Melampsora coleosporioides*. 8, *Puccinia phragmitis*. M, marker.

**Figure S8.** Characteristics of the translated protein of AvrLr15. (a) Predicted total amino acid sequence and signal peptide sequence (highlighted with rectangle) of the AvrLr15 protein. (b) The graphical output of SignalP v. 5.0 predictions.

**Figure S9.** Detection of recombinant plasmid pSUC2:SP_AvrLr15_ by PCR. (1) The amplification of pSUC2: SP_AvrLr15_. (2) The amplification of empty pSUC2 vector. M, marker.

**Figure S10.** Restriction digestion of recombinant plasmid pCamA:_ΔSP_AvrLr15. M, marker.

**Figure S11.** Fluorescence of the green fluorescent protein (GFP) fusion protein. The proteins were expressed in *Nicotiana benthamiana* following agroinfiltration. Fluorescence was detected in epidermal cells of the infiltrated leaves by fluorescence microscopy 48 h after agroinfiltration. Scale bars, 50 μm.

**Figure S12.** Polymorphisms of virulent *avrLr15* alleles were identified from naturally occurring *Lr15*‐breaking *Puccinia triticina* (Pt) isolates. Multiple sequence alignment of *AvrLr15* from 15 *Lr15* avirulent Pt isolates and avrLr15 from 10 *Lr15*‐breaking Pt isolates. The blue box indicated three amino acid mutations and one amino acid deletion at the P80, P92, M96 and P106 positions, respectively.

**Figure S13.** Restriction digestion of recombinant plasmid pCamA:_ΔSP_avrLr15. M, marker.

**Figure S14.** Expressed fluorescently tagged _ΔSP_avrLr15 location in the *Nicotiana benthamiana* leaves epidermal cells. The pCamA‐_ΔSP_avrLr15 (with GFP tag) in *N. benthamiana* cells and GFP field show green. Scale bar, 50 μm.

**Figure S15.**_ΔSP_avrLr15 can suppress BAX‐induced cell death. _ΔSP_avrLr15 was infiltrated into *Nicotiana benthamiana* leaves, followed 48 h later by infiltration with *Agrobacterium tumefaciens* carrying the *BAX* gene. pCamA and buffer served as a negative control.

**Figure S16.** Construction of mutants AvrLr15_P80A_, AvrLr15_ΔP92_, AvrLr15_M96L_, AvrLr15_P106S_, AvrLr15_P80A+ΔP92_, AvrLr15_M96L+P106S_.

**Figure S17.** The genomic alignment results of the *AvrLr15* sequence in 12 *Puccinia triticina* races. The races 60‐L‐2 and 64‐L‐3 are virulent to *Lr15*.

**Table S1.** Primers used in this study.

**Text S1.** Materials and methods.

## Data Availability

The gene sequence of *AvrLr15* is available in the National Center for Biotechnology Information (NCBI) (http://www.ncbi.nlm.nih.gov/) with accession number OQ458736.1.
