# Supplementary material for: Evasion of wheat resistance gene Lr15 recognition by the leaf rust fungus is attributed to the coincidence of natural mutations and deletion in AvrLr15 gene
Source: Mol Plant Pathol. 2024 Jul 2;25(7):e13490. doi: 10.1111/mpp.13490 (PMC11217590; doi:10.1111/mpp.13490)
Supplement: Supplementary file 1 — Figure S1. Detection of recombinant plasmid pGEX‐6P‐3:PTTG_27353 by PCR. (1) The amplification of empty pGEX‐6P‐3 vector; (2) the amplification of pGEX‐6P‐3:PTTG_27353; M, marker. [file MPP-25-e13490-s008.docx]

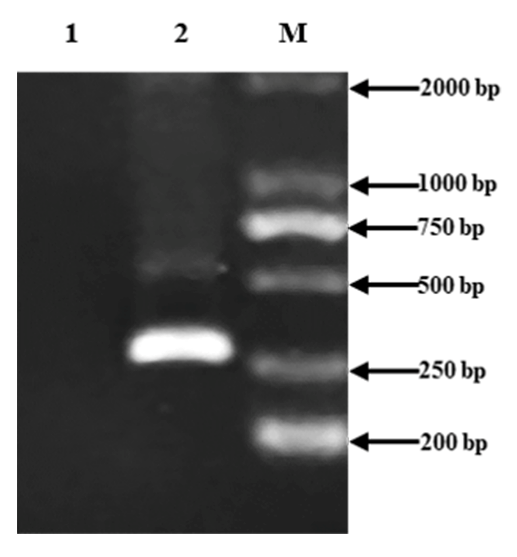


**Figure S1** Detection of recombinant plasmid pGEX-6P-3: PTTG_27353 by PCR. 1, The amplification of empty pGEX-6P-3 vector; 2, The amplification of pGEX-6P-3: PTTG_27353; M, Marker.
