# Supplementary material for: Evasion of wheat resistance gene Lr15 recognition by the leaf rust fungus is attributed to the coincidence of natural mutations and deletion in AvrLr15 gene
Source: Mol Plant Pathol. 2024 Jul 2;25(7):e13490. doi: 10.1111/mpp.13490 (PMC11217590; doi:10.1111/mpp.13490)
Supplement: Supplementary file 2 — Figure S2. Transient expression of PTTG_27353 induced a hypersensitive response in wheat TcLr15, a near‐isogenic line of Thatcher. Vector was used as a negative control for infiltration (left leaf of each pair); leaves on the right were infiltrated with PTTG_27353 protein. The red box pinpoints TcLr15. [file MPP-25-e13490-s019.docx]

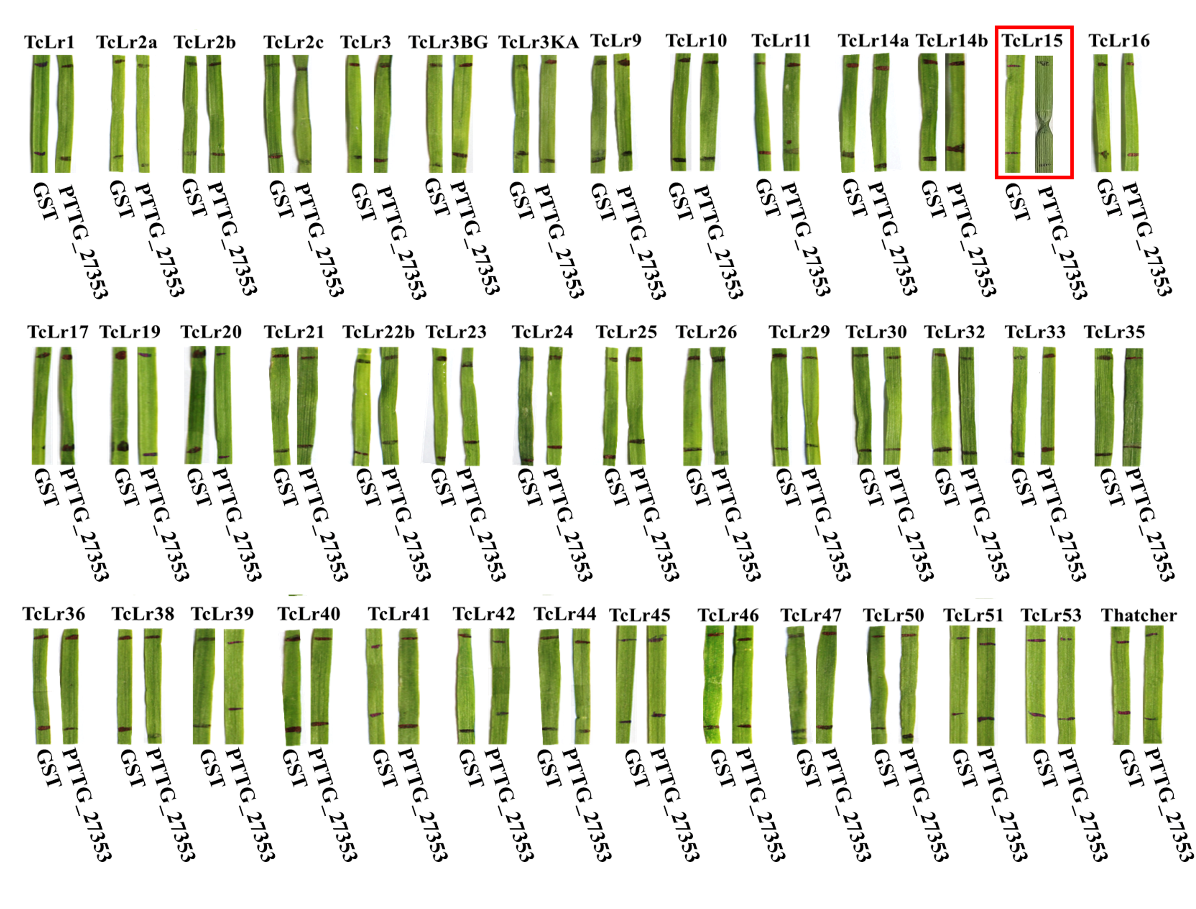


**Figure S2** Transient expression of *PTTG_27353* induced HR in wheat TcLr15, a near-isogenic line of Thatcher. Vector was used as a negative control for infiltration (left leaf of each pair); leaves on the right were infiltrated with PTTG_27353 protein. The red box pinpoints TcLr15.
