# Supplementary material for: Evasion of wheat resistance gene Lr15 recognition by the leaf rust fungus is attributed to the coincidence of natural mutations and deletion in AvrLr15 gene
Source: Mol Plant Pathol. 2024 Jul 2;25(7):e13490. doi: 10.1111/mpp.13490 (PMC11217590; doi:10.1111/mpp.13490)
Supplement: Supplementary file 3 — Figure S3. Silencing efficiency assay of AvrLr15 in AvrLr15‐silenced plants at 24, 48 and 120 hours post‐inoculation (hpi) with Puccinia triticina. Wheat leaves inoculated with empty vector BSMV:γ were used as controls. [file MPP-25-e13490-s002.docx]

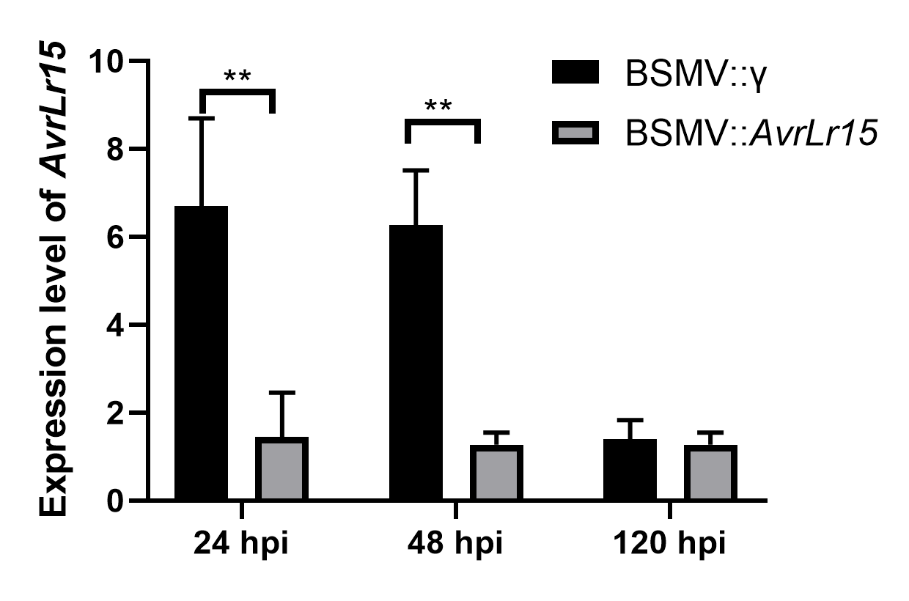


**Figure S3** Silencing efficiency assay of *AvrLr15* in AvrLr15-silenced plants at 24, 48 and 120hpi with *Pt*. Wheat leaves inoculated with BSMV: γ were used as controls
