# Supplementary material for: Evasion of wheat resistance gene Lr15 recognition by the leaf rust fungus is attributed to the coincidence of natural mutations and deletion in AvrLr15 gene
Source: Mol Plant Pathol. 2024 Jul 2;25(7):e13490. doi: 10.1111/mpp.13490 (PMC11217590; doi:10.1111/mpp.13490)
Supplement: Supplementary file 4 — Figure S4. Analysis nucleotide sequence of AvrLr15. (a) The red box indicates exons of AvrLr15. The green box indicates intron of AvrLr15. Black lines indicate untranslated region (UTR). Arrows indicate primer direction. (b) The red line indicates the exon sequence of the AvrLr15. The green line indicates the intron sequence of AvrLr15. [file MPP-25-e13490-s012.docx]

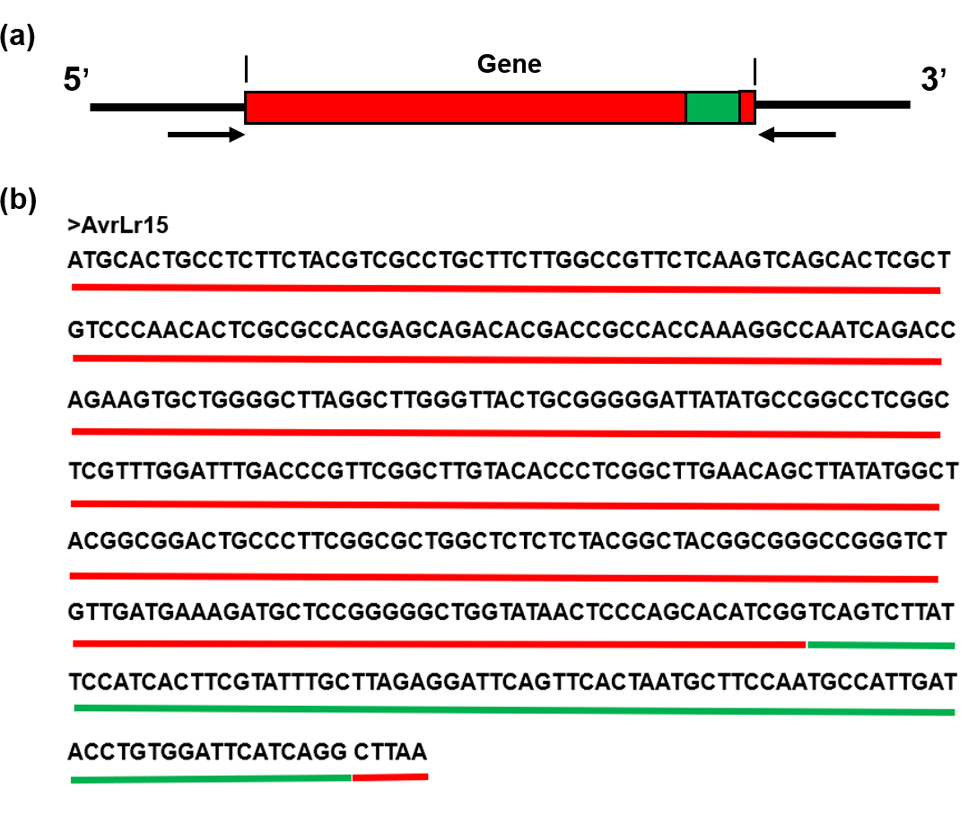


**Figure S4** Analysis nucleotide sequence of *AvrLr15*. (a) The red box indicates exons of *AvrLr15*. The green box indicates intron of AvrLr15. Black lines indicate UTR region. Arrows indicate primer direction; (b) The red line indicates the exon sequence of the *AvrLr15*. The green line indicates the intron sequence of *AvrLr15***.**
