# Supplementary material for: Evasion of wheat resistance gene Lr15 recognition by the leaf rust fungus is attributed to the coincidence of natural mutations and deletion in AvrLr15 gene
Source: Mol Plant Pathol. 2024 Jul 2;25(7):e13490. doi: 10.1111/mpp.13490 (PMC11217590; doi:10.1111/mpp.13490)
Supplement: Supplementary file 5 — Figure S5. PCR amplification products from genomic DNA of different Puccinia triticina races are shown after separation on a 1% agarose gel. 1, PHTT. 2, PHNT. 3, KHKT. 4, THKT. 5, RHHT. 6, THTT. 7, THTS. 8, sterile water. M, marker. [file MPP-25-e13490-s010.docx]

**Figure S5** PCR amplification products from genomic DNA of different *Pt* races are shown after separation on a 1% agarose gel. 1, PHTT. 2, PHNT. 3, KHKT. 4, THKT. 5, RHHT. 6, THTT. 7, THTS. 8, Sterile water. M, Marker.
