# Supplementary material for: Evasion of wheat resistance gene Lr15 recognition by the leaf rust fungus is attributed to the coincidence of natural mutations and deletion in AvrLr15 gene
Source: Mol Plant Pathol. 2024 Jul 2;25(7):e13490. doi: 10.1111/mpp.13490 (PMC11217590; doi:10.1111/mpp.13490)
Supplement: Supplementary file 6 — Figure S6. Multiple sequence alignment of AvrLr15 from different Puccinia triticina races. The red line indicates the exon sequence of the AvrLr15. The green line indicates the intron sequence of AvrLr15. [file MPP-25-e13490-s017.docx]

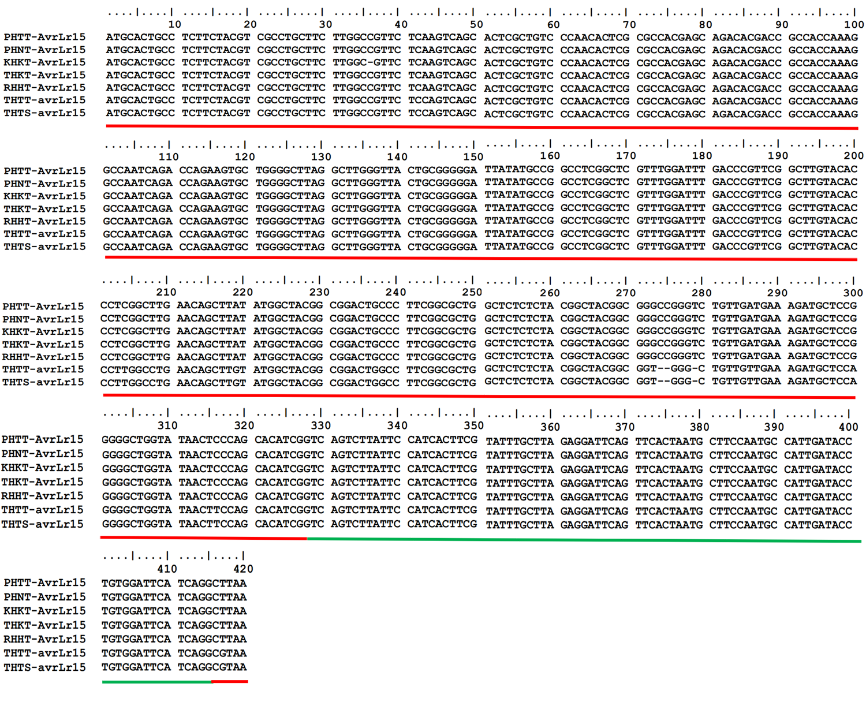


**Figure S6** Multiple sequence alignment of *AvrLr15* from different *Pt* races. The red line indicates the exon sequence of the *AvrLr15*. The green line indicates the intron sequence of *AvrLr15***.**
