# Supplementary material for: Evasion of wheat resistance gene Lr15 recognition by the leaf rust fungus is attributed to the coincidence of natural mutations and deletion in AvrLr15 gene
Source: Mol Plant Pathol. 2024 Jul 2;25(7):e13490. doi: 10.1111/mpp.13490 (PMC11217590; doi:10.1111/mpp.13490)
Supplement: Supplementary file 7 — Figure S7. PCR amplification products from different plant species rust fungi races are shown after separation on a 1% agarose gel. 1, Puccinia triticina. 2, Puccinia striiformis f. sp. tritici. 3, Puccinia graminis var. tritici. 4, Phakopsora ziziphivulgaris. 5, Gymnosporangium yamadai. 6, Uromyces verrucosus. 7, Melampsora coleosporioides. 8, Puccinia phragmitis. M, marker. [file MPP-25-e13490-s004.docx]

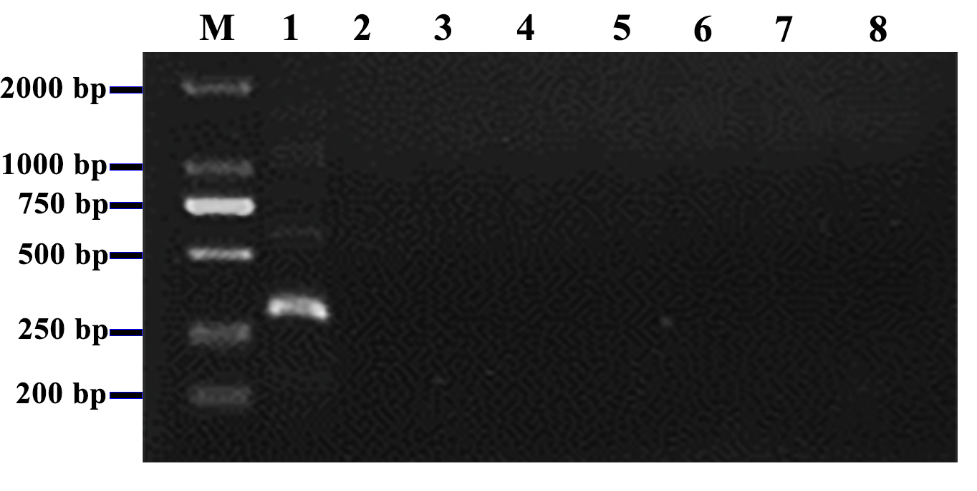


**Figure S7** PCR amplification products from different plant species rust fungi races are shown after separation on a 1% agarose gel. 1, *Puccinia triticina*. 2, *Puccinia striiformis f. sp. tritici*. 3, *Puccinia graminis Pers. var. tritici*. 4, *Phakopsora ziziphivulgaris (P. Henn.) Diet*. 5, *Gymnosporangium yamadai*. 6, *Uromyces verrucosus*. 7, *Melampsora coleosporioides*. 8, *Pucciniaphragmitis (Schum) Korn*. M, Marker.
