# Supplementary material for: Evasion of wheat resistance gene Lr15 recognition by the leaf rust fungus is attributed to the coincidence of natural mutations and deletion in AvrLr15 gene
Source: Mol Plant Pathol. 2024 Jul 2;25(7):e13490. doi: 10.1111/mpp.13490 (PMC11217590; doi:10.1111/mpp.13490)
Supplement: Supplementary file 8 — Figure S8. Characteristics of the translated protein of AvrLr15. (a) Predicted total amino acid sequence and signal peptide sequence (highlighted with rectangle) of the AvrLr15 protein. (b) The graphical output of SignalP v. 5.0 predictions. [file MPP-25-e13490-s015.docx]

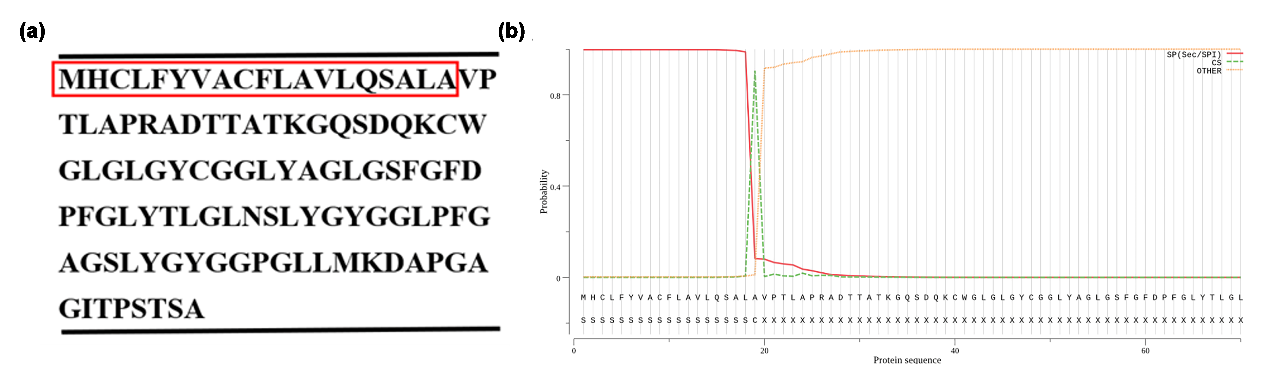


**Figure S8** Characteristics of the translated protein of AvrLr15. (a) Predicted total amino acid sequence and signal peptide sequence (highlighted with rectangle) of the AvrLr15 protein. (b) The graphical output of Signal P 5.0 predictions.
