# Supplementary material for: Evasion of wheat resistance gene Lr15 recognition by the leaf rust fungus is attributed to the coincidence of natural mutations and deletion in AvrLr15 gene
Source: Mol Plant Pathol. 2024 Jul 2;25(7):e13490. doi: 10.1111/mpp.13490 (PMC11217590; doi:10.1111/mpp.13490)
Supplement: Supplementary file 9 — Figure S9. Detection of recombinant plasmid pSUC2:SPAvrLr15 by PCR. (1) The amplification of pSUC2: SPAvrLr15. (2) The amplification of empty pSUC2 vector. M, marker. [file MPP-25-e13490-s007.docx]

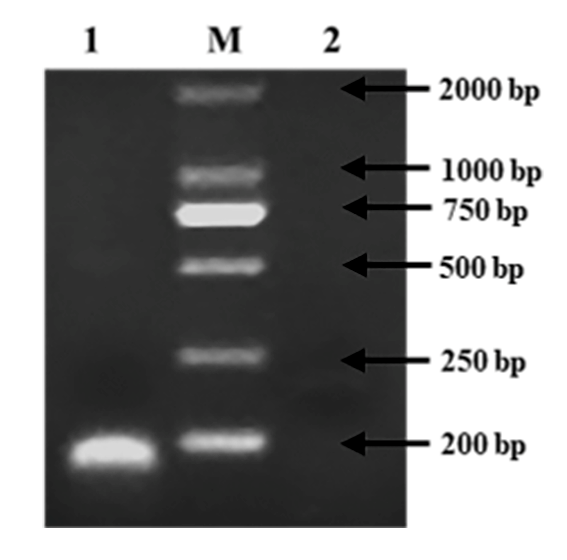


**Figure S9** Detection of recombinant plasmid pSUC2:SP_AvrLr15_ by PCR. 1, The amplification of pSUC2: SP_AvrLr15_; 2, The amplification of empty pSUC2 vector; M, Marker.
