# Supplementary figures and images for: Evasion of wheat resistance gene Lr15 recognition by the leaf rust fungus is attributed to the coincidence of natural mutations and deletion in AvrLr15 gene
Source: Mol Plant Pathol. 2024 Jul 2;25(7):e13490. doi: 10.1111/mpp.13490 (PMC11217590; doi:10.1111/mpp.13490)

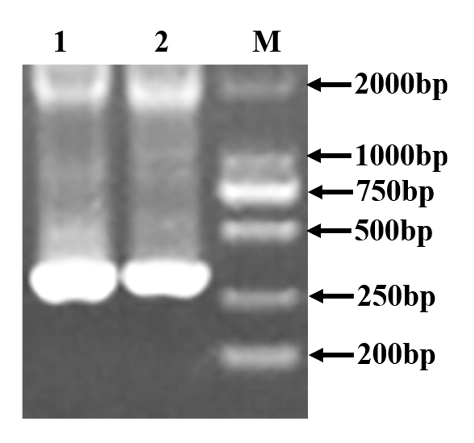


**Figure S10** Restrictive digestion of recombinant plasmid pCamA: △SPAvrLr15. M, Marker.

Supplement: Supplementary file 10 — Figure S10. Restriction digestion of recombinant plasmid pCamA:ΔSPAvrLr15. M, marker. [file MPP-25-e13490-s016.docx]

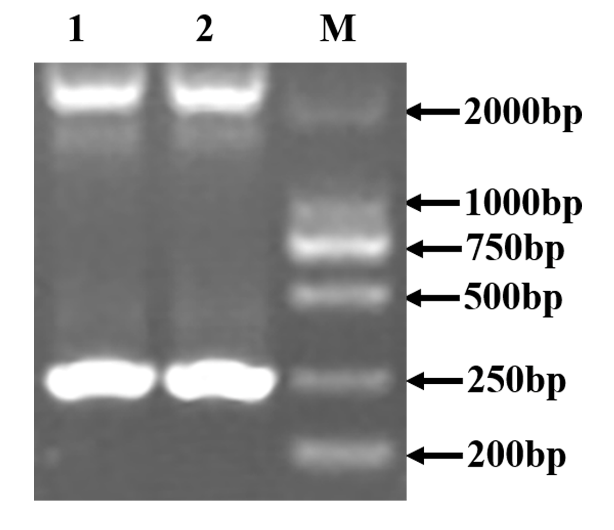


**Figure S13** Restrictive digestion of recombinant plasmid pCamA:△SPavrLr15. M, Marker.

Supplement: Supplementary file 13 — Figure S13. Restriction digestion of recombinant plasmid pCamA:ΔSPavrLr15. M, marker. [file MPP-25-e13490-s003.docx]

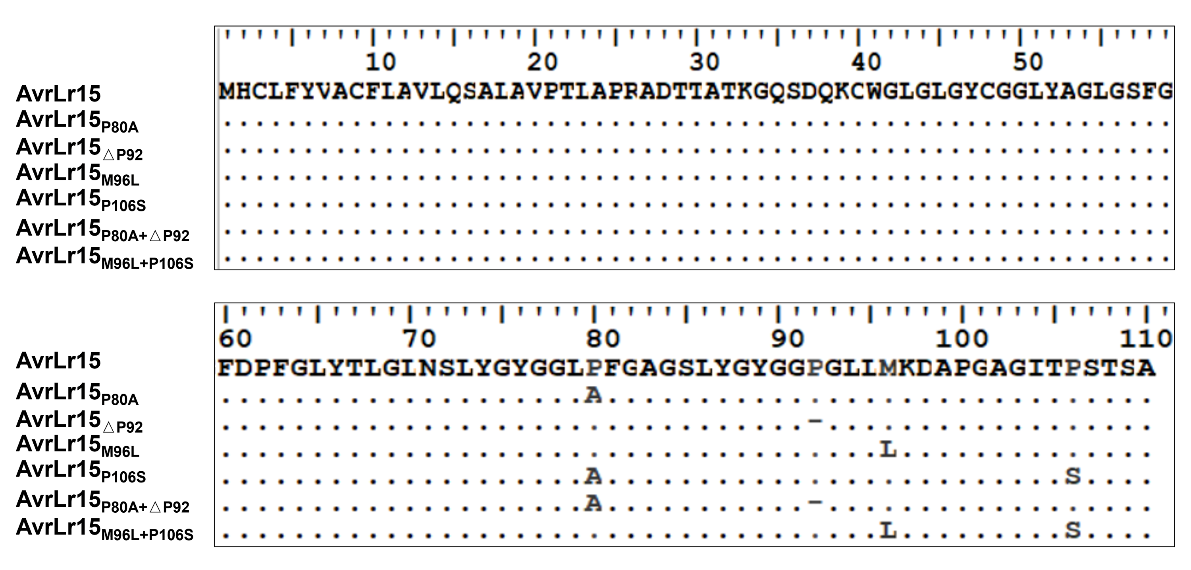


**Figure S16** Construction of mutants AvrLr15P80A, AvrLr15△P92, AvrLr15M96L, AvrLr15P106S, AvrLr15P80A+△P92, AvrLr15M96L+P106S.

Supplement: Supplementary file 16 — Figure S16. Construction of mutants AvrLr15P80A, AvrLr15ΔP92, AvrLr15M96L, AvrLr15P106S, AvrLr15P80A+ΔP92, AvrLr15M96L+P106S. [file MPP-25-e13490-s005.docx]
