# Supplementary material for: Evasion of wheat resistance gene Lr15 recognition by the leaf rust fungus is attributed to the coincidence of natural mutations and deletion in AvrLr15 gene
Source: Mol Plant Pathol. 2024 Jul 2;25(7):e13490. doi: 10.1111/mpp.13490 (PMC11217590; doi:10.1111/mpp.13490)
Supplement: Supplementary file 11 — Figure S11. Fluorescence of the green fluorescent protein (GFP) fusion protein. The proteins were expressed in Nicotiana benthamiana following agroinfiltration. Fluorescence was detected in epidermal cells of the infiltrated leaves by fluorescence microscopy 48 h after agroinfiltration. Scale bars, 50 μm. [file MPP-25-e13490-s006.docx]

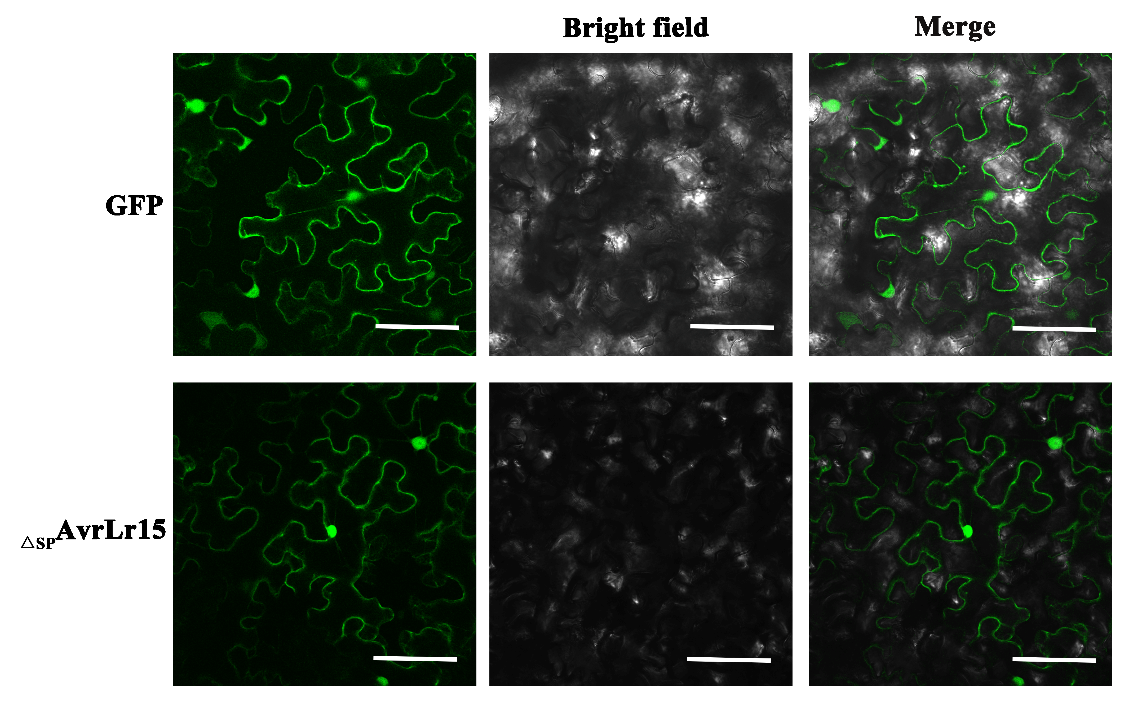


**Figure S11** Fluorescence of the GFP fusion protein. The proteins were expressed in *N. benthamiana* following agroinfiltration. Fluorescence was detected in epidermal cells of the infiltrated leaves by fluorescence microscopy 48 h after agroinfiltration. Scale bars, 50 μm.
