# Supplementary material for: Evasion of wheat resistance gene Lr15 recognition by the leaf rust fungus is attributed to the coincidence of natural mutations and deletion in AvrLr15 gene
Source: Mol Plant Pathol. 2024 Jul 2;25(7):e13490. doi: 10.1111/mpp.13490 (PMC11217590; doi:10.1111/mpp.13490)
Supplement: Supplementary file 12 — Figure S12. Polymorphisms of virulent avrLr15 alleles were identified from naturally occurring Lr15‐breaking Puccinia triticina (Pt) isolates. Multiple sequence alignment of AvrLr15 from 15 Lr15 avirulent Pt isolates and avrLr15 from 10 Lr15‐breaking Pt isolates. The blue box indicated three amino acid mutations and one amino acid deletion at the P80, P92, M96 and P106 positions, respectively. [file MPP-25-e13490-s018.docx]

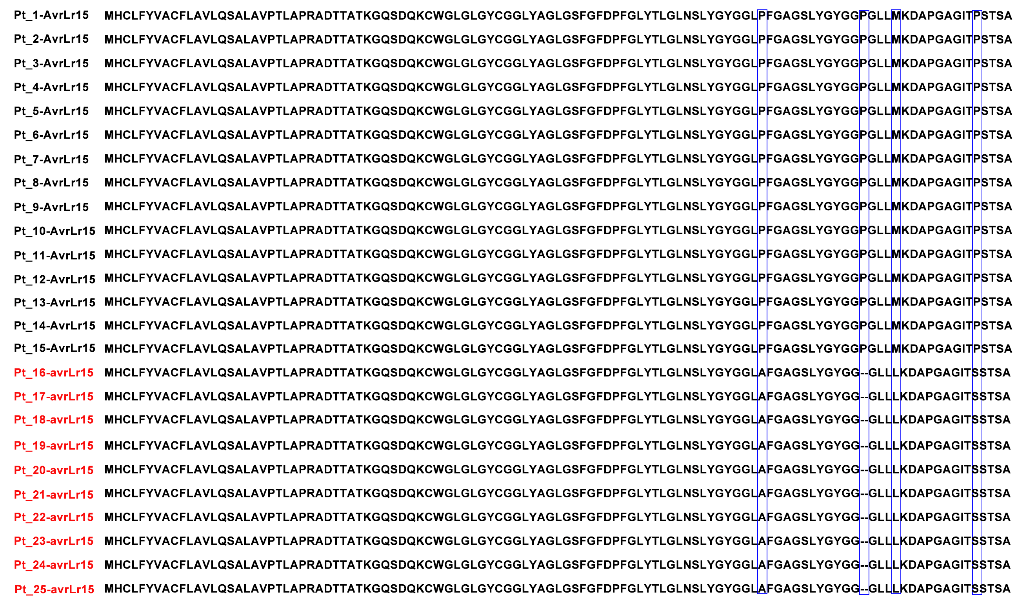


**Figure S12** Polymorphisms of virulent *avrLr15* alleles were identified from naturally occurring Lr15-breaking *Pt* isolates. Multiple sequence alignment of *AvrLr15* from fifteen *Lr15* avirulent *Pt* isolates and avrLr15 from ten *Lr15*-breaking *Pt* isolates. The blue box indicated three amino acid mutations and one amino acid deletion at the P80, P92, M96 and P106 positions, respectively.
