# Supplementary material for: Evasion of wheat resistance gene Lr15 recognition by the leaf rust fungus is attributed to the coincidence of natural mutations and deletion in AvrLr15 gene
Source: Mol Plant Pathol. 2024 Jul 2;25(7):e13490. doi: 10.1111/mpp.13490 (PMC11217590; doi:10.1111/mpp.13490)
Supplement: Supplementary file 14 — Figure S14. Expressed fluorescently tagged ΔSPavrLr15 location in the Nicotiana benthamiana leaves epidermal cells. The pCamA‐ΔSPavrLr15 (with GFP tag) in N. benthamiana cells and GFP field show green. Scale bar, 50 μm. [file MPP-25-e13490-s009.docx]

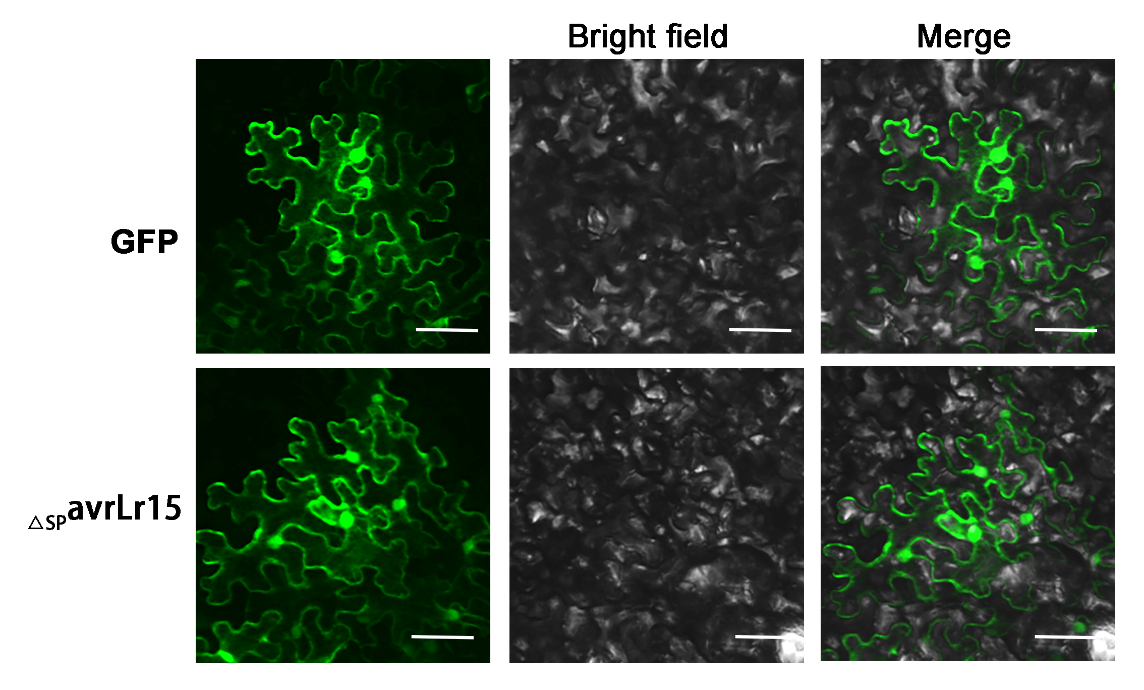


**Figure S14** Expressed fluorescently tagged _△SP_avrLr15 location in the *N. benthamiana* leaves epidermal cells. The pCamA-_△SP_avrLr15 (with GFP tag) in *N. benthamiana* cells and GFP field show green. Scale bar, 50 μm.
