# Supplementary material for: Evasion of wheat resistance gene Lr15 recognition by the leaf rust fungus is attributed to the coincidence of natural mutations and deletion in AvrLr15 gene
Source: Mol Plant Pathol. 2024 Jul 2;25(7):e13490. doi: 10.1111/mpp.13490 (PMC11217590; doi:10.1111/mpp.13490)
Supplement: Supplementary file 15 — Figure S15. ΔSPavrLr15 can suppress BAX‐induced cell death. ΔSPavrLr15 was infiltrated into Nicotiana benthamiana leaves, followed 48 h later by infiltration with Agrobacterium tumefaciens carrying the BAX gene. pCamA and buffer served as a negative control. [file MPP-25-e13490-s013.docx]

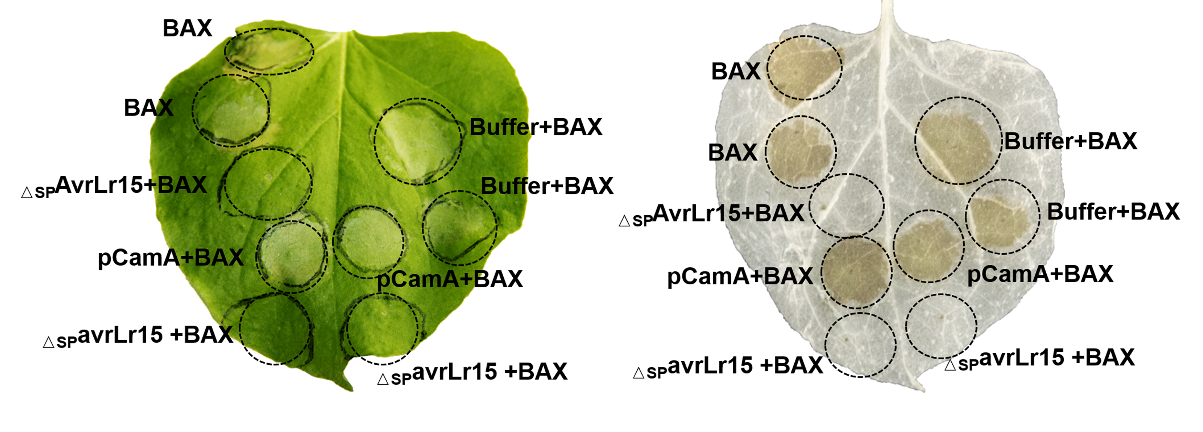


**Figure S15** _△SP_avrLr15 can suppress BAX-induced cell death. _△SP_avrLr15 was infiltrated into *N. benthamiana* leaves, followed 48 h later by infiltration with *A. tumefaciens* carrying the *BAX* gene. pCamA and buffer served as a negative control.
