# Supplementary material for: Evasion of wheat resistance gene Lr15 recognition by the leaf rust fungus is attributed to the coincidence of natural mutations and deletion in AvrLr15 gene
Source: Mol Plant Pathol. 2024 Jul 2;25(7):e13490. doi: 10.1111/mpp.13490 (PMC11217590; doi:10.1111/mpp.13490)
Supplement: Supplementary file 17 — Figure S17. The genomic alignment results of the AvrLr15 sequence in 12 Puccinia triticina races. The races 60‐L‐2 and 64‐L‐3 are virulent to Lr15. [file MPP-25-e13490-s001.docx]

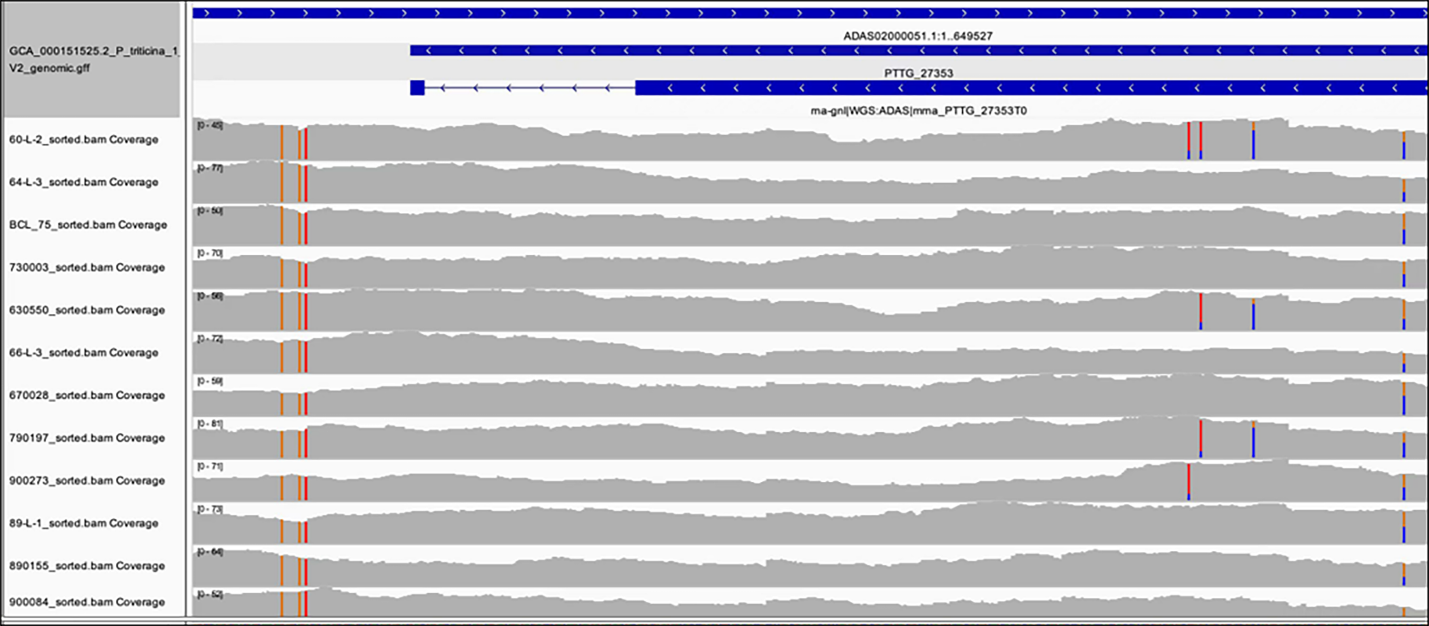


**Figure. S17** The genomic alignment results of the *AvrLr15* sequence in twelve *Pt* races. The races 60-L-2 and 64-L-3 are virulence to *Lr15*.
