# Supplementary material for: Evasion of wheat resistance gene Lr15 recognition by the leaf rust fungus is attributed to the coincidence of natural mutations and deletion in AvrLr15 gene
Source: Mol Plant Pathol. 2024 Jul 2;25(7):e13490. doi: 10.1111/mpp.13490 (PMC11217590; doi:10.1111/mpp.13490)
Supplement: Supplementary file 18 — Table S1. Primers used in this study. [file MPP-25-e13490-s014.docx]

**Table S1** Primers used in this study

| Primer name | Primer sequence |
| --- | --- |
| qPTTG-27353-F | GGGCTTAGGCTTGGGTT |
| qPTTG-27353-R | ATCAACAGACCCGGCCCGC |
| GST-PTTG_27353-F | GCCACGAGCAGACACGA |
| GST-PTTG_27353-R | GCCGATGTGCTGGGAGT |
| UTR-AvrLr15-F | ctctagaccaccaccgccacc |
| UTR-AvrLr15-R | cagcctaacaggatggtaagc |
| GST-AvrLr15-F | GCCACGAGCAGACACGA |
| GST-AvrLr15-R | GCCGATGTGCTGGGAGT |
| pCamA-AvrLr15-F | GCCACGAGCAGACACGA |
| pCamA-AvrLr15-R | GCCGATGTGCTGGGAGT |
| GST-avrLr15-F | GCCACGAGCAGACACGA |
| GST-avrLr15-R | GGTATAACTTCCAGCACATCG |
| pCamA-avrLr15-F | TGCCTTGCTCGTCTTGCTAA |
| pCamA-avrLr15-R | GGTATAACTTCCAGCACATCG |
| SP_AvrLr15_-F | ATGCACTGCCTCTTCTACG |
| SP_AvrLr15_-R | AGCGAGTGCTGACTTGA |
