# Supplementary material for: Evasion of wheat resistance gene Lr15 recognition by the leaf rust fungus is attributed to the coincidence of natural mutations and deletion in AvrLr15 gene
Source: Mol Plant Pathol. 2024 Jul 2;25(7):e13490. doi: 10.1111/mpp.13490 (PMC11217590; doi:10.1111/mpp.13490)
Supplement: Supplementary file 19 — Text S1. Materials and methods. [file MPP-25-e13490-s011.docx]

**Materials and methods**

**Plant materials, *Pt* race and sample collection**

All near-isogenic lines of Thatcher, susceptible wheat Chinese Spring and tobacco are preserved in the Laboratory of Wheat Leaf Rust, Hebei Agricultural University. Second seedling leaves inoculated with PHNT or distilled water (control) for RNA extraction were harvested at 0, 12, 24, 96, 144, 192, 264 and 360 hpi. All samples were immediately frozen in liquid nitrogen and stored at -80˚C. Each treatment included three independent biological replicates.

**RNA extraction and qPCR analysis**

Total RNA was extracted using M5 Plant RNeasy Complex Mini kit (Mei5bio, Beijing, China) according to the manufacturer’s instructions. cDNAs were synthesized using M5 Super Plus qPCR RT kit with gDNA remover (Mei5bio, Beijing, China). qPCR was conducted using 2×M5 HiPer Real-time PCR Super Mix (Mei5bio, Beijing, China) with an ABI QuantStudio 5 instrument (ABI, Waltham, Massachusetts, USA). The expression of *PTTG_27353* was investigated, the *β-actin* gene (GenBank accession OAV91054) was used to calibrate expression levels of queried genes. Data were analyzed by the 2^-ΔΔCT^ method (Livak & Schmittgen, 2002). The statistical significance of differences was calculated using one-way analysis of variance (ANOVA) and Duncan’s multiple range test (DMRT) with *p*<0.05 in SPSS 26.0 (IBM SPSS Statistics, IBM). For each treatment, three independent biological replicates were used for analysis.

**Sequence analysis**

*AvrLr15* was amplified from *Pt* race PHNT using 2×Es Taq MasterMix (CWBIO, Beijing, China). The molecular weight of AvrLr15 protein was predicted by ProtParam (<https://web.expasy.org/protparam/>). Effector protein prediction was conducted using EffectorP-fungi 3.0 (http://effectorp.csiro.au/). The signal peptide of AvrLr15 was predicted using SignalP 5.0 (http://www.cbs.dtu.dk/services/SignalP/). All primers used in this study are listed in Table S1.

**Expression and purification of the fusion protein**

*AvrLr15*, *avrLr15* were individually cloned into pGEX-6p-3 vector (with GST tag). The recombinant plasmids were transformed into *E. coli* BL21 (DE3). The proteins were induced using previously published method (Salcedo *et al*, 2017). A GST-Agarose Label Kit (TRAN, Beijing, China) was used to bind GST tagged protein following the manufacturer’s instructions. The purified protein products were separated by 15% SDS-PAGE and visualized by Coomassie Blue staining.

**Infiltration of wheat near-isogenic lines**

We infiltrated 21 days-old seedlings of all available near-isogenic lines of Thatcher with AvrLr15 protein. This protein was diluted to 0.1 mg/ml in 1×PBS buffer (137 Mm NaCl, 2.7 Mm KCl, 10 Mm Na_2_HPO_4_, and 2 Mm KH_2_PO_4_) and infiltrated into the abaxial side of the first leaf using 1 ml syringe. Buffer served as a negative control. AvrLr15, avrLr15 were purified and infiltrated into TcLr15. Infiltrated seedlings were transferred to growth chamber maintained at 22°C with photoperiod of 16 h. Cell death was evaluated at 24 h after infiltration. Genomic DNA of TcLr15 (RL6052) was extracted following a previously published method (Ortiz *et al*, 2022).

***Agrobacterium* infiltration assay**

*Agrobacterium*-mediated transient transformation of *N. benthamiana* was performed according to methods described previously (Oh *et al*, 2009). Briefly, *Agrobacterium* cultures containing _ΔSP_AvrLr15 expression vectors were grown overnight at 28°C in LB media with appropriate antibiotic selections. The cells were pelleted and resuspended in buffer to an optical density (OD_600_) of 0.6. Infiltrations were conducted on leaves of 4-5-week old tobacco. The resulting responses were photographed at 4 days after infiltration. Suppression of BAX-triggered cell death by effector _ΔSP_AvrLr15 was determined by the *Agrobacterium*-mediated transient expression method. The infiltration sites (_ΔSP_AvrL15) were challenged 24 h later with an *Agrobacterium* culture carrying BAX at a final OD_600_ of 1. Symptoms were monitored and recorded from 2 to 4 days after infiltration.

**Yeast secretion trap assay**

The yeast signal sequence trap system was used to validate the signal peptide function of AvrLr15 as described previously (Ma *et al*, 2012). The signal peptide sequence of *AvrLr15* was cloned into vector pSUC2T7M13ORI (pSUC2) using specific primers (Table S1) and then transformed into invertase mutant yeast strain YTK12. CMD-W medium was used to screen the positive colonies. The secretory function was confirmed by growth on YPRAA plates containing raffinose and lacking glucose. Invertase activity was detected by the reduction of 2,3,5-triphenyltetrazolium chloride (TTC) to insoluble red-colored 1,3,5-triphenylformazan (TPF) according to procedures and conditions described previously (Ma *et al*, 2012).

**Host-induced gene silencing (HIGS)**

*In vitro* transcription was performed using the mMESSAGE mMACHINE Kit High Yield Capped RNA Transcription Kit (Ambion) with linearized plasmid as template according to the manufacturers protocol. Then 240 μl of the BSMV mixture was applied to fully expanded leaves of 4-leave stage TcLr15 wheat plants by rubbing according to the method described previously (Zhang *et al*, 2018). Leaf rust urediniospores were inoculated on the fourth and fifth leaves of TcLr15 and the susceptible wheat cultivar Thatcher. Sterile water was inoculated to wheat leaves as control. The more details of HIGS assay were performed according to a previous study (Panwar et al, 2013). The feasibility and silencing efficiency were tested using the wheat phytoene desaturase (TaPDS) as a positive control.

**DNA amplification**

Genomic DNA from different *Pt* races was extracted according to previously published method^34^. AvrLr15 sequences were amplified from genomic DNA using primers in Table S1. PCR products were sequenced directly.

**References**

Livak, K.J., & Schmittgen, T.D. (2002) Analysis of relative gene expression data using real-time quantitative PCR and the 2^-ΔΔCT^ method. *Methods*, 25, 402-408.

Ma, L., Lukasik, E., Gawehns, F., & Takken, F.L. (2012) The use of agroinfiltration for transient expression of plant resistance and fungal effector proteins in *Nicotiana benthamiana* leaves. *Methods in Molecular Biology*, 835, 61-74.

Oh, S.K., Young, C., Lee, M., Oliva, R., Bozkurt, T.O., Cano, L.M., Win, J., Bos, J.I., Liu, H.Y., van Damme M. et al. (2009) *In planta* expression screens of *Phytophthora infestans* RXLR effectors reveal diverse phenotypes, including activation of the *Solanum bulbocastanum* disease resistance protein Rpi-blb2, *Plant Cell*, 21, 2928-2947.

Ortiz, D., Chen, J., Outram, M.A., Saur, I.M.L., Upadhyaya, N.M., Mago, R.et al. (2022) The stem rust effector protein AvrSr50 escapes Sr50 recognition by a substitution in a single surface exposed residue. *New Phytologist*, 234, 592-606.

Panwar, V., McCallum, B. & Bakkeren, G. (2013) Host-induced gene silencing of wheat leaf rust fungus *Puccinia triticina* pathogenicity genes mediated by the barley stripe mosaic virus. *Plant Molecular Biology*, 81, 595–608.

Salcedo, A., Rutter, W., Wang, S., Akhunova, A., Bolus, S., Chao, S. et al. (2017) Variation in the *AvrSr35* gene determines *Sr35* resistance against wheat stem rust race Ug99. *Science*, 358, 1604-1606.

Zhang, J., Wang, F., Liang, F., Zhang, Y., Ma, L., Wang, H., & Da, Q. (2018) Functional analysis of a pathogenesis-relatedthaumatin-like protein gene *TaLr35PR5* from wheat induced by leaf rust fungus. *BMC Plant Biology*, 18, 76-87.
